# Effectiveness of a Postpartum Breastfeeding Support Group Intervention in Promoting Exclusive Breastfeeding and Perceived Self-Efficacy: A Multicentre Randomized Clinical Trial

**DOI:** 10.3390/nu16070988

**Published:** 2024-03-28

**Authors:** Isabel Rodríguez-Gallego, Isabel Corrales-Gutierrez, Diego Gomez-Baya, Fatima Leon-Larios

**Affiliations:** 1 Foetal Medicine, Genetics and Reproduction Unit, Virgen del Rocío University Hospital, 41013 Seville, Spain; isroga@cruzroja.es; 2 Red Cross Nursing University Centre, University of Seville, 41009 Seville, Spain; 3 Surgery Department, Faculty of Medicine, University of Seville, 41009 Seville, Spain; 4 Foetal Medicine Unit, Virgen Macarena University Hospital, 41009 Seville, Spain; 5 Department of Social, Developmental and Educational Psychology, Universidad de Huelva, 21007 Huelva, Spain; 6 Nursing Department, School of Nursing, Physiotherapy and Podiatry, University of Seville, 41009 Seville, Spain; fatimaleon@us.es

**Keywords:** breastfeeding, lactation, exclusive breastfeeding, self-efficacy, breastfeeding support groups, community health services, lactation support, midwifery, public health, randomized controlled trial

## Abstract

There are numerous recognized benefits of breastfeeding; however, sociocultural, individual, and environmental factors influence its initiation and continuation, sometimes leading to breastfeeding rates that are lower than recommended by international guidelines. The aim of this study was to evaluate the effectiveness of a group intervention led by midwives supporting breastfeeding during the postpartum period in promoting exclusive breastfeeding, as well as to assess the impact of this intervention on perceived self-efficacy. This was a non-blind, multicentric, cluster-randomized controlled trial. Recruitment started October 2021, concluding May 2023. A total of 382 women from Andalusia (Spain) participated in the study. The results showed that at 4 months postpartum there was a higher prevalence of breastfeeding in the intervention group compared to formula feeding (*p* = 0.01), as well as a higher prevalence of exclusive breastfeeding (*p* = 0.03), and also at 6 months (*p* = 0.01). Perceived self-efficacy was similar in both groups for the first two months after delivery, which then remained stable until 4 months and decreased slightly at 6 months in both groups (*p* = 0.99). The intervention improved the average scores of perceived self-efficacy and indirectly caused higher rates of exclusive breastfeeding (*p* = 0.005). In conclusion, the midwife-led group intervention supporting breastfeeding proved to be effective at maintaining exclusive breastfeeding at 6 months postpartum and also at increasing perceived self-efficacy.

## 1. Introduction

The World Health Organization (WHO) advocates for breastfeeding as an unparalleled method of feeding that can provide all the nutrients a newborn needs for growth and immunological development in the first months of life. Breastfeeding provides half or more of a child’s nutritional requirements during the second semester of life and up to a third during the second year [1]. Therefore, the current recommendation by the WHO, along with the United Nations Children’s Fund, is that breast milk should be the exclusive food for newborns until the age of 6 months and that, until the age of 2 years, they should be fed a combination of breast milk and age-appropriate, nutritious foods [2].

There is increasing scientific evidence of the multiple benefits that breastfeeding brings the newborn, at physical, cognitive, and psychosocial levels [1,2,3,4], as well as the mother, by preventing pathologies related to physical and mental health [5,6,7,8]. In fact, there are numerous comprehensive reviews that summarize the benefits of breastfeeding and the mechanisms by which these are achieved by describing a series of increasingly well-understood complex pathways through which breast milk has evolved to optimize child survival. Even in recent epidemics, such as that caused by severe acute respiratory syndrome coronavirus, breastfeeding has been demonstrated to be superior to other types of infant feeding [9,10]. However, the global prevalence of breastfeeding indicates that, although the initiation of breastfeeding occurs in almost all countries, there is a progressive decrease in the number of mothers who continue breastfeeding over the first few months of a newborn’s life [11,12,13].

In today’s society, both social and cultural determinants, as well as support from health services, family and community support, social policies and work-life balance, and individual factors related to maternal and child health, influence the initiation and continuation of breastfeeding [14]. Specifically, the promotion and support of breastfeeding immediately after birth, skin-to-skin contact, avoidance of separating the newborn from the mother, and community support are prognostic factors for the success of breastfeeding [15,16].

Recent studies also indicate that group support interventions have a greater impact on breastfeeding rates than individual counselling. Prenatal advice has a positive effect, achieving better breastfeeding rates at 4–6 weeks postpartum, while the combination of prenatal and postnatal advice favours the prolongation of breastfeeding up to 6 months. Therefore, both prenatal and postnatal counselling and support are recommended to achieve better breastfeeding rates [17]. Furthermore, other studies [18,19,20] have shown that peer support has a greater effect on the initiation, maintenance, and duration of breastfeeding when led by professionals, reinforcing the idea of midwife-led group support interventions for breastfeeding mothers.

Various individual maternal factors, such as attitudes and expectations regarding breastfeeding and a lack of confidence in breastfeeding, can be modified through educational interventions during pregnancy and postpartum support. Maternal lack of confidence in breastfeeding is a point highlighted by mothers themselves when they discuss their experience and is an important predictor of premature cessation of breastfeeding [21]. Studies have shown that maternal self-efficacy in breastfeeding is a modifiable factor that can improve breastfeeding rates [22,23].

Numerous studies of environmental factors and, more specifically, work-life balance at the national and international levels [15,24,25,26] indicate that existing policies are insufficient, with the return to work being one of the main reasons for the early cessation of breastfeeding (before 6 months). Specifically, in Spain, maternity leave and leave for childcare generally last 16 weeks, which is less than the WHO’s recommendation for exclusive breastfeeding [27].

The principal aim of this study was to evaluate the effectiveness of a midwife-led group intervention supporting breastfeeding during the postpartum period in promoting exclusive breastfeeding up to when newborns reached 6 months of age. The secondary objective was to assess the effect of this intervention on breastfeeding self-efficacy and its relationships with the duration and exclusivity of breastfeeding.

## 2. Materials and Methods

### 2.1. Study Setting

This was a multicentric cluster-randomized controlled trial with a control group and an intervention group and was not blinded. It was completed as described in our published protocol [28]. In addition, the trial was registered in the International Standard Registered Clinical/Social Study Number registry (Trial ID: ISRCTN17263529; date recorded: 17 June 2020).

### 2.2. Participants and Study Area

Eligible female participants were recruited in primary health centres in Andalusia, Spain. Andalusia is an autonomous community divided into eight provinces with a total of 8,472,407 inhabitants (data available in 2021) [29] and a birth rate of 7.72 per 1000 inhabitants (2021) [30]. By 1 July 2022, the number of women of reproductive age in Andalusia was 4,328,407 [31]. The study was conducted on the populations from the provinces of Seville, Cadiz, Huelva, Granada, and Jaen.

According to data provided by the National Statistical Institute of Spain, in 2021, there were a total of 65,650 births in Andalusia. Births in the provinces of Seville (15,655), Granada (7083), Huelva (4227), Jaen (4499), and Cadiz (8904) totalled 40,368, which represented 61.79% of the total births in the community [32]. 

### 2.3. Sample Design

The rate of exclusive breastfeeding at 6 months in Andalusia is 39% [33], which was estimated as the expected value in the control group. An estimated increase of 10%, as indicated by previous studies [14,34], in the rates of exclusive breastfeeding at 6 months was established. To achieve this difference between the two groups, a two-tailed hypothesis was posed, with a power of 80% and allowing for a type I error of 5%. The necessary sample size amounted to 371 women distributed between the two study groups.

### 2.4. Inclusion and Exclusion Criteria

Inclusion criteria:

1. Healthy women performing exclusive or partial breastfeeding 10 days after birth who attended antenatal lessons at the primary health centre.
2. Women over 18 years of age.
3. Women who accepted and signed the informed consent form.

Exclusion criteria:

1. Human immunodeficiency virus-positive.
2. Cancer.
3. Tuberculosis infection.
4. No intention to breastfeed.
5. Impossibility or contraindication to breastfeed due to medical conditions.
6. Premature and/or complicated labour or newborn in a neonatal intensive care unit during the first month of life.
7. Communication difficulties due to language barriers.

### 2.5. Randomization

Primary health centres were randomized into an intervention group or control group (usual care), considering whether any type of group breastfeeding support intervention was already being conducted there. The research technician assigned to the project, independent of the researchers who oversaw participant recruitment, performed this health centre allocation using a random sequence generated by the Oxford Minimization and Randomization system [35]. The technician assigned random unique identifiers to the health centres, differentiating between centres belonging to the control and intervention groups. Finally, out of a total of 23 primary health centres, 11 were included, 6 in the IG (2 centres in Seville, 1 centre in Huelva, 1 centre in Granada, 1 centre in Jaén, and 1 centre in Cádiz) and 5 in the CG (one centre per province). Each centre had a designated lead midwife responsible for recruiting participants and conducting the intervention, in the case of the intervention group.

After the randomization of centres for the recruitment of women into the intervention and control groups, each participant was assigned an identification code depending on the group to which she belonged.

### 2.6. Study Intervention

Women in the control group received the usual care regarding maternal education from the 28th week of pregnancy onward and postpartum visits, according to the Protocol for Care during Pregnancy, Childbirth, and Puerperium by the Andalusian Health and Social Welfare Council [36]. In the first 10 days after childbirth, they had an individual visit with the midwife, during which a breastfeeding session was observed using the WHO breastfeeding observation sheet [37], and concerns were resolved individually. Women also had the option of requesting on-demand individual postpartum consultations with the reference midwife of their health centre. All of this is included in standard care.

In addition, women in the intervention group received the usual prenatal and postpartum care, just like the control group. Subsequently, they participated in monthly 2 h face-to-face and/or virtual group sessions called breastfeeding support groups, for which the midwife acted as leader and moderator. These sessions had an educational component, through theoretical-practical presentations related to breastfeeding, based on the recommendations of the Baby-Friendly Hospital Initiative [38]. They also had a motivational component and a component based on the social or peer support established in the group. Thus, monthly, women were offered support by an organized and proactive professional. In addition to monthly meetings, participants had the option to interact with each other, with other breastfeeding women, and with the reference midwife via a Facebook™ and/or WhatsApp™ group established for this purpose. Thus, peer support was reinforced, and questions about the topic were resolved using information and communication technologies [39]. Likewise, participating women had the option of requesting on-demand individual consultations with the reference midwife, the same as women receiving usual care.

### 2.7. Instrument with Validity and Reliability

The study collected the following data: the participant’s sociodemographic information (age, level of education, marital status, employment, ethnicity); obstetric outcomes (home labour and delivery, mode of delivery, gestation weeks); and neonatal outcomes (sex, weight, Apgar, neonatology admission, health problems). Incorrect or incomplete data were corrected via direct consultation with participants or were collected from their health medical records with their consent.

In relation to breastfeeding, outcomes were collected for previous experience in breastfeeding (multiparous women were asked about their experience with breastfeeding while raising previous children, as well as the reason for giving it up); the type of breastfeeding during the follow-up; and, in cases of interruption, the reason.

The types of breastfeeding were classified according to [15]:

- Exclusive breastfeeding: the newborn is fed only with breast milk, without using any other milk or food, from its birth up until the first 6 months of its corrected age.
- Partial breastfeeding: occasional administration of formula milk.
- Mixed breastfeeding: combination of breast and formula milk.
- Artificial breastfeeding: exclusively formula milk.

Breastfeeding self-efficacy was measured using a reduced version of the Breastfeeding Self-efficacy Scale-Short Form (BSES-SF), which was validated in Spanish by Oliver-Roig et al. [40]. This scale is a structured questionnaire that measures maternal confidence through 14 items grouped in only one dimension. The items are positively presented and preceded by the phrase ‘I can always…’. Scoring is by a Likert-type scale from 1–5, where 1 indicates ‘not sure at all’ and 5 indicates ‘very sure’. Higher scores indicate higher self-efficacy levels for breastfeeding. The reliability of the Spanish version of the BSES-SF, as measured with the Cronbach alpha coefficient, was 0.92.

### 2.8. Data Collection

Participant recruitment began in October 2021 and ended in May 2023. It was conducted by the midwives responsible for each health centre, who received prior training on the project and were also advised by a research technician midwife of the project who was not involved in the execution of the intervention. 

The referring midwife of each health centre, in a consultation of week 35–37 to all women who met the established inclusion criteria, was informed of the nature and objectives of the study, as well as of the follow-ups to be carried out. In addition, at the postpartum visit, it was verified that the woman met the criteria for partial or exclusive breastfeeding at 10 days. Once the women agreed to participate, they signed the informed consent form in duplicate. Participants provided information through the web application project created for the study, which automatically sent them a reminder message and an email at the three assessment moments designed in the study.

The main control and outcome variables were collected before the start of the intervention (baseline) and at the 2–4- and 6-month follow-ups. The data relating to electronic follow-ups were coded and safeguarded by the research team. All data were stored in an electronic database accessible only to members of the research team.

### 2.9. Data Analysis

The analysis was conducted according to the intention-to-treat principle, regardless of whether participants adhered to the requirement to participate in the breastfeeding support group. The individual health centres were the randomization unit, and mother-infant dyads were the unit of analysis. All statistical tests and confidence intervals used a type I error rate set at alpha = 0.05 and were conducted using the SPSS v. 23 [41] statistical package (IBM).

First, an exploratory analysis of the different variables studied was performed. For the descriptive analysis of categorical variables, frequency distribution tables and percentages were generated. For continuous variables, means and standard deviations were calculated. Second, differences between the control and intervention groups in all descriptive study variables were analysed. The relationship between two categorical variables was analysed by developing contingency tables using Pearson’s chi-square test. For continuous variables, to examine differences between two groups, the independent samples Student’s *t*-test was conducted. To examine differences in continuous variables between three groups, ANOVA was used.

Third, the effectiveness analysis was conducted by comparing the proportion of women exclusively breastfeeding at 6 months in both groups using the McNemar test. Contingency tables were designed to examine the percentage of breastfeeding mothers based on whether or not they were in the intervention group. These tables were used to analyse the percentages before and after the intervention and after follow-up. To analyse changes in breastfeeding self-efficacy, a repeated measures ANOVA, controlling for the intervention group and the control group at the three evaluation time points of the study, was conducted. Additionally, the association between postpartum breastfeeding type and breastfeeding type after follow-up was examined using a chi-square test. Finally, the relationship between type of breastfeeding and employment status was examined using a chi-square test.

To examine the extent to which the use of breastfeeding after the intervention could be explained by the increase in breastfeeding self-efficacy, a partial mediation model was designed. In this model, based on regression analysis, the intervention acted as the independent variable (x), self-efficacy as the mediator (m), and breastfeeding as the dependent variable (y). The standardized coefficients of the model were analysed, as was the change in the total effect of the intervention on breastfeeding after including the mediating mechanism. These analyses were performed according to the method proposed by Hayes et al. [42] with the PROCESS macro v. 4.1 (2022) in SPSS v. 28.1 for Windows (IBM Corp. 2018, Armonk, NY, USA).

### 2.10. Ethical Considerations

Participation in the project was voluntary, as was the participation request. Verbal and written informed consent information was provided to every participant in the study. The study was designed according to the Spanish regulation act No. 14/2007 of 3 July regarding biomedical research, and complied with the study suitability requirements and with the procedures regarding the study objectives. All patient-related data collected for this study were treated according to the Spanish Organic Law on Protection of Personal Data and Guarantee of Digital Rights (Spanish Organic Law 3/2018).

The study was approved by the Research Ethics Committees of the Virgen Macarena and Virgen del Rocío hospitals (Seville, Spain) on 24 February 2020 (Code 1936-N-19).

## 3. Results

### 3.1. Characteristics of the Sample

A total of 382 women participated in the study: 232 (60.5%) in the intervention group and 151 (39.5%) in the control group. Table 1 shows the main sociodemographic and obstetric characteristics of the participants. At the recruitment baseline, the average age of all participants was 33.41 (standard deviation (SD) = 4.66) years, with the majority being of Spanish nationality (93.45%), having a university education (64.39%), being employed (71.46%), and predominantly working less than 20 h per week (25.39%). In terms of obstetric characteristics, most births were of spontaneous onset (60.47%), had eutocic delivery (61.78%), and occurred on average at 39.45 (SD = 1.25) weeks of gestation. Approximately 51.04% of the newborns were male, with an average weight of 3262.58 g (SD = 463.76). Most participants (57.85%) had no previous breastfeeding experience. No statistically significant differences in any of these characteristics were observed between the groups, indicating a homogeneous sample (Table 1).

**Figure 1:**
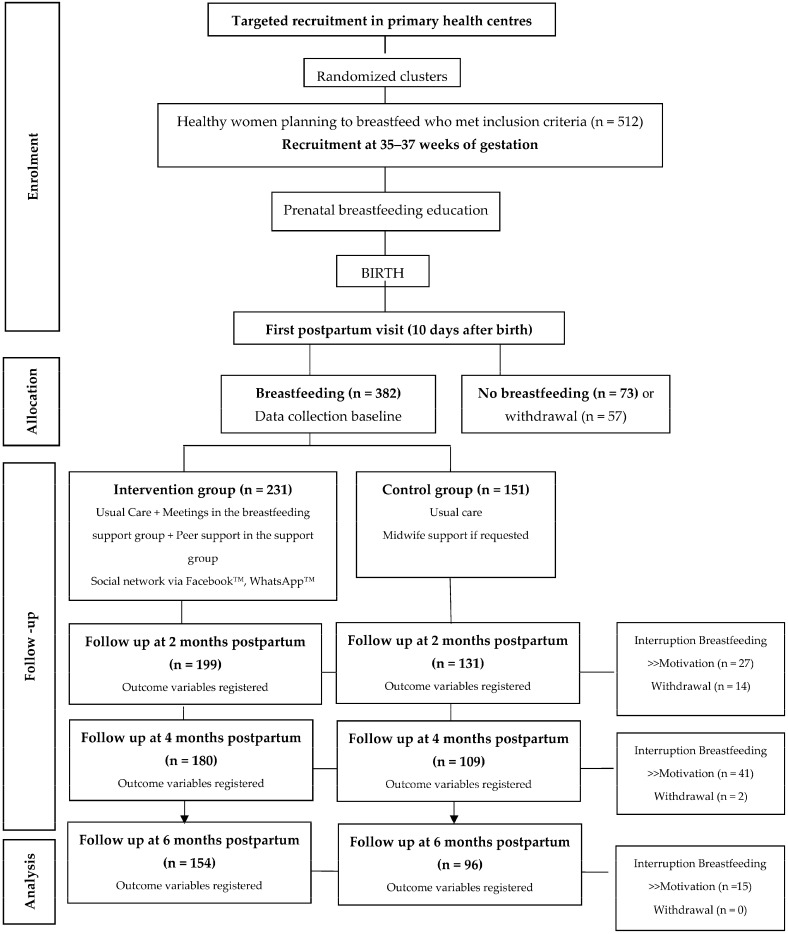
Participant selection flowchart.

The dropout rate at the 4-month follow-up was similar in both groups (intervention group = 12.38% vs. control group = 17.8%; *p* = 0.81; Figure 1).

### 3.2. Effectiveness of the Intervention in Influencing the Type of Breastfeeding

From the start of breastfeeding to 6 months postpartum, higher rates of breastfeeding, specifically exclusive breastfeeding, were maintained over time in the intervention group than in the control group, and breastfeeding rates were considerably higher from 2 months postpartum onward.

At 2 months postpartum, 89.3% of women in the control group continued with breastfeeding of various types, compared to 93.5% of women in the intervention group (χ^2^ (3) = 2.60, *p* = 0.44, Cramer’s V = 0.10). However, at 4 months postpartum, when attendance at support groups had accumulated in the intervention group, a statistically significant difference in the prevalence of breastfeeding was observed between groups (intervention group = 88.9% vs. control group = 80.7%) compared to formula feeding, which was significantly higher in the control group (intervention group = 11.1% vs. control group = 19.3%; χ^2^ (3) = 13.19, *p* < 0.01, Cramer’s V = 0.24).

On the other hand, at 6 months postpartum, 95.2% of women continued breastfeeding, compared to 90.7% of participants in the control group. Especially different were the percentages of mixed breastfeeding at this time point between both groups (intervention group = 21.4% vs. control group = 31.3%; χ^2^ (3) = 7.02, *p* = 0.07, Cramer’s V = 0.17).

Table 2 shows the comparison of the type of breastfeeding between the intervention and control groups during the follow-up period up to 6 months postpartum.

### 3.3. Effectiveness of the Intervention in Promoting Exclusive Breastfeeding

At the start of the study, similar percentages of exclusive breastfeeding were observed in both groups, with slightly higher rates in women who received only routine care (intervention group = 77.9% vs. control group = 78.1%; χ^2^ (1) = 0.002, *p* = 0.96, V = 0.002). However, after continued attendance of breastfeeding support groups by women in the intervention group, at 4 months postpartum, significant differences begin to be observed. A total of 69.4% of women in the intervention group were exclusively breastfeeding, compared to 51.4% in the control group (χ^2^ (1) = 8.72, *p* = 0.03, V = 0.15). Similarly, at 6 months postpartum, 63.4% of women in the intervention group continued exclusive breastfeeding, compared to 47.9% of women in the control group (χ^2^ (1) = 5.98, *p* = 0.01, V = 0.15).

Supplementary Table S1 shows changes in the type of breastfeeding from the start of the postpartum period to 2, 4, and 6 months later in both study groups. 

At two months postpartum, 86.5% of women who initiated exclusive breastfeeding continued with this type of feeding in the intervention group, compared to 72.8% of women in the control group (χ^2^ (3) = 10.11, *p* < 0.01, V = 0.20). On the other hand, among women in the control group who exclusively breastfed at the beginning of the study, 57.5% continued exclusive breastfeeding at 4 months, while 11.5% continued with occasional help, 16.1% opted for mixed feeding, and 14.9% abandoned breastfeeding in favour of formula. However, in the intervention group, 78.5% of women who started with exclusive breastfeeding continued with this type of feeding until 4 months postpartum, and only 4.9% abandoned breastfeeding (χ^2^ (3) = 16.8, *p* < 0.01, V = 0.26). At six months postpartum, a significant trend was observed towards a shift from occasional breastfeeding assistance to exclusive breastfeeding in the intervention group compared to the control (intervention group = 57.1% vs. control group = 8.3%; χ^2^ (3) = 8.81, *p* = 0.03, V = 0.58).

### 3.4. Breastfeeding Self-Efficacy

**Figure 2:**
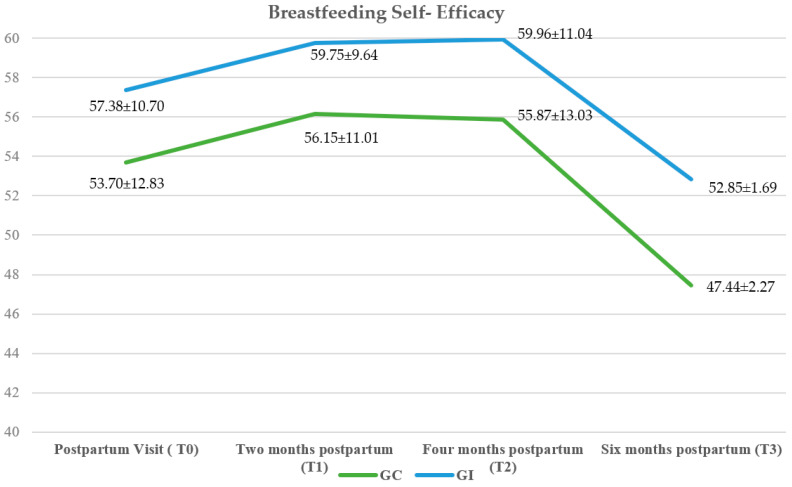
Changes in breastfeeding self-efficacy over time.

At the start of the study, women in the intervention group had slightly higher average perceived breastfeeding self-efficacy scores than those in the control group (intervention group = 57.38 ± 10.70 vs. control group = 53.70 ± 12.83). From the start to 2 months postpartum, a slight increase in self-efficacy was observed (intervention group = 59.75 ± 9.64 vs. control group = 56.15 ± 11.01), after which scores remained similar until 4 months postpartum (intervention group = 59.96 ± 11.04 vs. control group = 55.87 ± 13.03). However, at 6 months postpartum, a decrease in perceived self-efficacy was observed in both groups, this being more pronounced in women who received usual care (intervention group = 52.85 ± 1.69 vs. control group = 47.44 ± 2.27). There were no statistically significant differences in breastfeeding self-efficacy between the two study groups (F (3) = 0.19, *p* = 0.99) (Figure 2).

**Figure 3:**
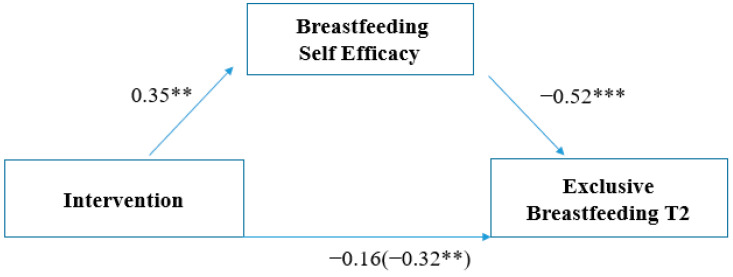
Mediational role of perceived breastfeeding self-efficacy on the relationship between intervention and exclusive breastfeeding at 4 months postpartum. Note: ** significant *p*-values < 0.01; *** significant *p*-values < 0.001.

Table 3 shows the results of the mediation analysis of the relationship between the intervention and exclusive breastfeeding at 4 months postpartum (where the highest ratio was observed) through the effects of breastfeeding self-efficacy. Figure 3 shows the standardized coefficients of the relationships included in the model. The results indicated that the effect of the intervention on exclusive breastfeeding at 4 months postpartum was fully mediated by the indirect effect of breastfeeding self-efficacy. The total effect of the intervention (before including the mediator) was β = −0.32 (*p* = 0.002), and it reduced to β = −0.16 (*p* = 0.78) after including the mediating variable of breastfeeding self-efficacy. Thus, the analysis revealed that the intervention improved average perceived self-efficacy scores, and, indirectly, this increase contributed to higher rates of exclusive breastfeeding, particularly at 4 months postpartum (F (2, 271) = 89.12, *p* < 0.001, R^2^ = 0.47).

### 3.5. Early Cessation of Breastfeeding 

At 2 months postpartum, 13 participants (6.5%, z = −1.6) who attended the support groups prematurely abandoned breastfeeding, compared to 14 women (10.7%, z = 1.6) in the control group. The main reasons reported globally for this cessation were a feeling of low milk production (37.3%), weight loss in the newborn (33.3%), difficulty latching (22.2%), and difficulty with breastfeeding practices (7.40%) (χ^2^ (1) = 2.50, *p* = 0.11, V = 0.08).

In contrast, at 4 months postpartum, the observed percentages of early breastfeeding cessation were nearly double in the control group (*n* = 21, 19.30%, z = 1.9) compared to the intervention group (*n* = 20, 11.1%, z = −1.9). At this time point, the main reasons for early cessation were returning to work (34.78%), difficulty latching (17.39%), weight loss in the newborn (15.21%), a feeling of low milk production (13.04%), mastitis (10.81%), and personal desire (8.69%) (χ^2^ (1) = 8.49, *p* = 0.04, V = 0.17).

At 6 months postpartum, 7 women (4.5%; z = −1.5) in the intervention group chose to discontinue breastfeeding in favor of formula feeding, compared to 9 women (9.4%; z = 1.5) in the control group. The reasons for discontinuation were returning to work (43.75%), introduction of complementary feeding (43.75%), and personal desire (12.5) (χ^2^ (1) = 2.30, *p* = 0.12, V = 0.96).

### 3.6. Employment and Breastfeeding

Participants in the intervention group who continued with their paid maternity leave at 4 months postpartum had higher percentages of exclusive breastfeeding (78.25%) than women in the same situation who received only standard care (50%). Additionally, women in the control group in the same employment situation more often stopped breastfeeding prematurely and had higher percentages of formula feeding (25%) than in the intervention group (10.3%) (χ^2^ (3) = 11.66, *p* = 0.09, V = 0.29).

Similarly, among women who returned to work, a higher percentage of them continued with exclusive breastfeeding at 4 months postpartum in the intervention group (69.2%) than in the control group (44.4%). In women in the control group, a greater inclination towards formula feeding (22.2%) and, therefore, early cessation of breastfeeding, was observed (χ^2^ (3) = 10.95, *p* = 0.12, V = 0.37; Table 4).

At 6 months postpartum, only 13.6% of women (*n* = 34) were still on paid maternity leave, compared to 57.2% (*n* = 143) of women who were employed. A notable difference was observed in the percentages of exclusive breastfeeding among employed women by study group (intervention group = 60% vs. control group = 45.1%), as well as for other types of breastfeeding (χ^2^ (3) = 5.53, *p* = 0.13, V = 0.2; Table 5).

## 4. Discussion

This multicentric cluster-randomized controlled trial aimed to analyse the impacts of a midwife-led group intervention that supported breastfeeding during the postpartum period and evaluate its effectiveness in promoting exclusive breastfeeding until newborns reached 6 months of age. Additionally, this study assessed the effect of this intervention on breastfeeding self-efficacy and its relationships with the duration and exclusivity of breastfeeding. This study provided evidence that additional support for routine breastfeeding care, in the form of a midwife-led group intervention with peer support, was an effective intervention that improved breastfeeding rates up to 6 months postpartum. Specifically, the designed intervention demonstrated that women who received additional support showed a relative increase of about 20% in exclusive breastfeeding rates at 4 months and a relative increase about 15% at 6 months postpartum. This key finding agrees with other studies that reported similar results [43,44]: exclusive breastfeeding increased in the intervention group when community-based interventions were conducted, including counselling or group support, immediate breastfeeding support during childbirth and postpartum, and breastfeeding management. The main increase in these rates was observed from 2 months postpartum onward, with the intervention becoming significantly effective at 4 months postpartum. This result was also observed in a study by Moudi et al. [45], which compared a routine care group with two experimental groups, one that received peer support and one that received support from healthcare providers.

In addition to the face-to-face support of the group, participants received online support through groups created on social networks that reinforced the main intervention, favoured peer support, and facilitated quick and safe access to information [46,47]. This strategy has been demonstrated by other studies [48,49,50] to be effective in maintaining exclusive breastfeeding rates when investigated as a single support component. However, in the present clinical trial, combined face-to-face and online support was provided, following the recommendations of recent meta-analyses [19,51] that have advocated for a multicomponent intervention involving a health professional as an effective strategy to improve global breastfeeding and exclusive breastfeeding. This recommended intervention involves theory taught face-to-face and subsequent online follow-up during the prenatal and postnatal periods.

With the rise of new technologies and the impact of the 2019 coronavirus disease pandemic on health services, social network support for breastfeeding, in addition to being effective, has become increasingly necessary, popular, and important for women [52]. This type of support is an indispensable resource, not only for providing information and solving breastfeeding-related problems, but also for emotional support [53]. Online support has also been shown to be effective in promoting attitudes related to breastfeeding [54], increasing the levels of breastfeeding self-efficacy of participating women [55,56], one of the main findings of the present study.

Additionally, this clinical trial confirmed that the effectiveness of support groups was enhanced by the effect of the intervention on perceived breastfeeding self-efficacy. The observed average scores were higher in all follow-up periods in the intervention group than for the women who received only routine support. Other studies [57,58,59] have reported similar findings for the intervention group and have also observed higher scores in mothers who breastfed exclusively than in those who did not. Franco-Antonio et al. [60] already observed, in their clinical trial on the effect of a brief motivational intervention conducted immediately postpartum, that higher breastfeeding self-efficacy scores predicted the durations of both exclusive and non-exclusive breastfeeding. This relationship between high self-efficacy and prolonged exclusive breastfeeding is partially explained by a study by Blyth et al. [61], in which the main finding was that mothers with higher self-efficacy were more likely to adapt and react more positively to breastfeeding difficulties and, therefore, were more likely to continue breastfeeding. Unlike other factors, breastfeeding self-efficacy is a potentially modifiable individual determinant that can improve breastfeeding rates, as reported by previous studies [59,62,63,64,65], when combined with interventions by health professionals [23,66,67]. Moreover, breastfeeding self-efficacy has been recognized as one of the factors positively associated with the establishment and duration of exclusive breastfeeding [68,69], even in premature newborns [70,71], and it has also been identified as a significant predictor of breastfeeding after future pregnancies [72]. These findings provide additional evidence that support aimed at improving breastfeeding rates also improves self-efficacy expectations and, therefore, the probability of successful exclusive breastfeeding.

In this study, which was conducted in the same country as the LACTEM [15] study in 2016, similar results were found on the subjective sensation of low milk production, which was one of the main reasons for the early cessation of breastfeeding. Additionally, other international studies [73] have shown this factor to be one of the most prevalent among those driving the cessation of breastfeeding. For example, Colin et al. [74] observed that this sensation, in addition to promoting the cessation of breastfeeding, generated substantial anxiety among mothers and that many of them experienced this anxiety for up to 6 months postpartum, inclusive. Another relevant factor promoting the cessation of breastfeeding is insufficient weight gain of the newborn, which emerged as an important maternal concern in a study by Odom et al. [75].

Previous national and international [76,77,78] studies have found a correlation between women’s return to work and lower breastfeeding rates and, specifically, exclusive breastfeeding rates. The present study found similar results that, although not statistically significant, might have clinical relevance. However, women who attended the support groups showed higher breastfeeding rates at 6 months, as did participants in other studies who received additional support or resources, such as a favourable environment [79] or a support network [80].

There are some limitations of this study. First, there is a limitation in relation to the number of participants per research group, because the recruitment of women from the control group was more difficult because there was no hypothetical benefit related to the study and there was no blinding of the participants. For this reason, they declined to participate in the study in greater numbers. On the other hand, although a population may share the same nationality, it is crucial to recognize intra-cultural and health system differences that may influence the results of a study. Therefore, the importance of replicating studies in different contexts to validate and generalize the findings is emphasized, thus ensuring a more complete and accurate understating of the phenomena studied. Second, the success of the intervention may have been modulated by mothers’ predispositions to participate in such groups and receive additional support. Additionally, adherence to group attendance may have been influenced by the degree of leadership shown by the midwife who guided and moderated the group, although all received prior training involving attitudes and knowledge. Third, the trial dropout rate by the 6-month follow-up was over 15%, although the sample size was adequate at the time of recruitment, the reasons for withdrawal were recorded, and the method of data collection was easy and accessible to women at any time and from any electronic device, without the need for travel. Fourth, data related to breastfeeding and self-efficacy were self-reported, which could introduce a memory or desirability bias. Finally, the 2019 coronavirus disease pandemic affected the follow-up of participants and ability to conduct the intervention in person, as instructions from the Ministry of Health changed due to variation in the incidence and prevalence of the disease.

In future research, a more personalized follow-up is suggested to prevent losses during the follow-up and the possibility of offering the control group a health intervention unrelated to breastfeeding could be considered to increase participation. On the other hand, it would also be interesting to explore the informal support received by the participants during the study, as well as whether there are differences between online and face-to-face groups, in order to offer results adjusted to the intervention modality. It would be advisable to monitor the long-term impact of the intervention, beyond the recommended period of exclusive breastfeeding. This way, results regarding the impact of the intervention on prolonged breastfeeding and the introduction of complementary feeding could be provided. The subjective results reported by the participants can be accompanied by objective observations made by midwives or detailed feeding diaries. Furthermore, assessing the satisfaction of women attending midwife-led support groups through focus groups would provide valuable insights.

## 5. Conclusions

Breastfeeding support groups, a group intervention led by midwives and aimed at supporting breastfeeding during the postpartum period, proved to be effective at maintaining exclusive breastfeeding up to 6 months postpartum. Additionally, the intervention improved perceived breastfeeding self-efficacy, which is a modifiable factor, so the effectiveness of this intervention was mediated by higher self-efficacy scores in women who attended the support groups.

One of the main factors for the early cessation of breastfeeding at 4 and 6 months postpartum, return to work, can be combated with additional face-to-face and online support in the form of support groups. However, additional measures are needed to attain the breastfeeding rates recommended by international organizations.

Once the effectiveness of this midwife-led group intervention is demonstrated, guidelines could be developed for professionals describing the implementation practices of this support resource. These findings should encourage a shift in the current breastfeeding support system towards an integrated network of support led by midwives to achieve improved maternal and child health. 

## Figures and Tables

**Table 1:** Comparison of principal sociodemographic and obstetric characteristics of the groups.

Characteristic	Total (*n* = 382)	Group		χ^2^	*t*	*p*-Value
		Intervention (*n* = 231)	Control (*n* = 151)			
Baseline						
Maternal age, years; mean ± SD	33.41 ± 4.66	33.50 ± 4.41	33.28 ± 5.03		−0.45	0.64
Nationality, *n* (%)				0.14		0.70
Spanish	357 (93.45)	215 (93.07)	142 (94.03)			
Other	25 (6.54)	16 (6.92)	9 (5.96)			
Education level, *n* (%)				0.81		0.84
Without	1 (0.26)	1 (0.4)	0 (0)			
Primary studies	11 (3.66)	9 (3.9)	5 (3.3)			
Secondary studies	121 (31.67)	74 (32)	47 (31.1)			
University studies	246 (64.39)	147 (63.6)	99 (65.6)			
Employment, *n* (%)				2.08		0.35
Employed	273 (71.46)	165 (71.4)	108 (71.5)			
Unemployed	109 (28.53)	66 (28.6)	43 (28.5)			
Work hours (per week), *n* (%)				34.46		0.44
<20	97 (25.39)	57 (34.5)	40 (37.0)			
20–30	75 (19.63)	44 (40.7)	31 (28.7)			
>20	57 (14.92)	33 (20)	24 (22.2)			
Gestation, weeks; mean ± SD	39.45 ± 1.25	39.45 ± 1.14	39.46 ± 1.38		0.13	0.89
Home labour and delivery (%)				0.13		0.71
Induced	151 (39.52)	61 (40.4)	90 (59.6)			
Spontaneous	231 (60.47)	89 (38.5)	142 (61.5)			
Mode of delivery, *n* (%)				2.88		0.41
Eutocic	236 (61.78)	140 (60.6)	96 (63.6)			
Dystocic	77 (20.15)	51 (22.1)	26 (17.2)			
SCS	17 (4.45)	12 (5.2)	5 (3.3)			
UCS	52 (13.61)	28 (12.1)	24 (15.9)			
Infant sex, *n* (%)				0.16		0.68
Male	195 (51.04)	116 (50.2)	79 (52.3)			
Female	187 (48.95)	115 (49.8)	72 (47.7)			
Newborn weight, g; mean ± SD	3262.58 ± 463.76	3239.10 ± 483.92	3298.51 ± 430.19		1.22	0.22
Previous breastfeeding experience, *n* (%)				1.42		0.23
Yes	161 (42.14)	103 (44.6)	58 (38.4)			
No	221 (57.85)	128 (55.4)	93 (61.6)			

Note: χ^2^, chi-square test; *t*, independent samples *t*-test; significant *p*-values < 0.05; SD, standard deviation; SCS, scheduled Caesarean section; UCS, urgent Caesarean section.

**Table 2:** Comparison of type of breastfeeding between the intervention and control groups.

Type of Breastfeeding	Time Postpartum							
	T0		T1		T2		T3	
	Group		Group		Group		Group	
	IG *n* (%)	CG *n* (%)	IG *n* (%)	CG *n* (%)	IG *n* (%)	CG *n* (%)	IG *n* (%)	CG *n* (%)
Exclusive breastfeeding	180 (77.9)	118 (78.1)	144 (72.4)	85 (64.9)	125 (69.4)	56 (51.4)	98 (63.4)	46 (47.9)
*z*-value	−0.1	0.1	1.4	−1.4	3.1	−3.1	2.4	−2.4
Breastfeeding with occasional help	31 (13.4)	20 (13.2)	18 (9)	14 (10.7)	22 (11.7)	12 (11.0)	16 (10.4)	11 (11.5)
*z*-value	0.0	0.0	−0.5	0.5	0.2	−0.2	−0.3	0.3
Breastfeeding mixed	20 (8.7)	13 (8.6)	24 (12.1)	18 (13.7)	14 (7.8)	20 (18.3)	32 (21.4)	30 (31.3)
*z*-value	0.0	0.0	−0.4	0.4	−2.7	2.7	−1.7	1.7
Artificial breastfeeding	--	--	13 (6.5)	14 (10.7)	20 (11.1)	21 (19.3)	7 (4.5)	9 (9.4)
*z*-value			−1.3	1.3	−1.9	1.9	−1.5	1.5

Note: T0, postpartum; T1, 2 months postpartum; T2, 4 months postpartum; T3, 6 months postpartum IG, intervention group; CG, control group.

**Table 3:** Results of the partial mediation model.

					95% Confidence Interval	
		β	*t*	*p*	Lower	Upper
Direct effect	Intervention ➝ Exclusive breastfeeding T2	−0.16	−1.77	0.78	−0.34	−0.18
Total effect	Intervention ➝ Exclusive breastfeeding T2	−0.32	−3.15	0.00	−0.53	−0.12
Effect on mediator	Intervention ➝ Breastfeeding self-efficacy	0.35	2.77	0.005	0.10	0.60
Effect by mediator	Breastfeeding self-efficacy ➝ Exclusive breastfeeding T2	−0.52	−12.55	<0.001	−0.60	−0.44

Note: T2, 4 months postpartum.

**Table 4:** Breastfeeding type by employment at 4 months postpartum.

Employment	Type of Breastfeeding								χ^2^ (*df*)	*p*-Value	*V*
	Exclusive Breastfeeding (%)		Breastfeeding with Occasional Help (%)		Breastfeeding Mixed (%)		Artificial Breastfeeding (%)				
	Group		Group		Group		Group				
	IG *n* (%)	CG *n* (%)	IG *n* (%)	CG *n* (%)	IG *n* (%)	CG *n* (%)	IG *n* (%)	CG *n* (%)			
Paid maternity leave	58 (77.3)	28 (50.9)	4 (5.3)	6 (10.9)	5 (6.7)	8 (14.5)	8 (10.7)	13 (23.6)	9.90 (3)	0.01	0.28
*z*-value	3.1	−3.1	−1.2	1.2	−1.5	1.5	−2.0	2.0			
Active duty mothers	36 (69.2)	11 (52.4)	10 (19.2)	2 (9.5)	3 (5.8)	5 (23.8)	3 (5.8)	3 (14.3)	6.7 (3)	0.07	0.31
*z*-value	−0.1	−1.5	1.9	−0.9	−0.4	2.2	−1.4	1.2			
Unemployed	31 (62)	17 (56.7)	8 (16)	4 (13.3)	6 (12)	7(23.3)	5 (10)	2(6.7)	1.9 (3)	0.6	0.15
*z*-value	0.5	−0.5	0.3	−0.3	−1.3	1.3	0.5	−0.5			

Note: χ^2^, chi-square test; *df*, degrees of freedom; V, Cramer’s V; significant *p*-values < 0.05; IG, intervention group; CG, control group.

**Table 5:** Breastfeeding type by employment at 6 months postpartum.

Employment	Type of Breastfeeding								χ^2^ (*df*)	*p*-Value	*V*
	Exclusive Breastfeeding (%)		Breastfeeding with Occasional Help (%)		Breastfeeding Mixed (%)		Artificial Breastfeeding (%)				
	Group		Group		Group		Group				
	IG *n* (%)	CG *n* (%)	IG *n* (%)	CG *n* (%)	IG *n* (%)	CG *n* (%)	IG *n* (%)	CG *n* (%)			
Paid maternity leave	17 (89.5)	10 (66.7)	--	3 (20)	2 (10.5)	2 (13.3)	--	--	4.40 (2)	0.11	0.36
*z*-value	1.6	−1.6	−2.0	2.0	−0.3	0.3	--	--			
Active duty mothers	56 (60.0)	23 (45.1)	11 (12)	5 (9.8)	21 (22.8)	17 (33.3)	4 (4.3)	6 (11.8)	5.55 (3)	0.13	0.2
*z*-value	1.8	−1.8	0.4	−0.4	−1.4	1.4	−1.7	1.7			
Unemployed	24 (60)	13 (44.8)	5 (12.5)	3 (10.3)	8 (20)	10 (34.5)	3 (7.5)	3 (10.3)	2.3 (3)	0.51	0.18
*z*-value	1.2	−1.2	0.3	−0.3	1.4	1.4	−0.4	0.4			

Note: χ^2^, chi-square test; *df*, degrees of freedom; *V*, Cramer’s *V*; significant *p*-values < 0.05; IG, intervention group; CG, control group.

## Data Availability

The data presented in this study are available on request from the corresponding author. The data are not publicly available due to confidentiality issues.

## Supplementary Materials

The following supporting information can be downloaded at: https://www.mdpi.com/article/10.3390/nu16070988/s1, Table S1. Breastfeeding type postpartum to 2, 4 and 6 months postpartum.
