# Supplementary material for: Effectiveness of a Postpartum Breastfeeding Support Group Intervention in Promoting Exclusive Breastfeeding and Perceived Self-Efficacy: A Multicentre Randomized Clinical Trial
Source: Nutrients. 2024 Mar 28;16(7):988. doi: 10.3390/nu16070988 (PMC11013075; doi:10.3390/nu16070988)
Supplement: Supplementary file 1 [file nutrients-16-00988-s001.zip › nutrients-2924115-supplementary.pdf]

Supplementary Table S1: Breastfeeding type postpartum to 2,4 and 6 months postpartum.

Total type of breast-feeding postpartum	Type of breastfeeding at 2 months postpartum										
	**Exclusive breastfeeding**		**Breastfeeding with occasional help**		**Breastfeeding mixed**		**Artificial breastfeeding**		**χ^2 (df)**	***p-Value***	***V***
	**Group**		**Group**		**Group**		**Group**				
	**IG *n* (%)**	**CG *n* (%)**	**IG *n* (%)**	**CG *n* (%)**	**IG *n* (%)**	**CG *n* (%)**	**IG *n* (%)**	**CG *n* (%)**			
Exclusive breastfeeding (n=298)	135(86.5)	75(72.8)	11(7.1)	9(8.7)	8(5.1)	13(12.6)	2(1.3)	6(5.8)	10.11(3)	<0.01	0.20
z-value	2.8	-2.8	-0.5	0.5	-2.2	2.2	-2.1	2.1			
Breastfeeding with occasional help(n=51)	5(18.5)	7(38.9)	5(18.5)	5(27.8)	10(37)	2(11.1)	7(25.9)	4(22.2)	4.88(3)	0.18	0.32
z-value	-1.5	1.5	-0.7	0.7	1.9	-1.9	0.3	-0.3			
Breastfeeding mixed (n=33)	4(25)	3(30)	2(12.5)	--	6(37.5)	3(30)	4(25)	4(40)	1.85(3)	0.6	0.26
z-value	-0.3	0.3	1.2	-1.2	0.4	-0.4	-0.8	0.8			

Total type of breast-feeding postpartum	Type of breastfeeding at 4 months postpartum										
	**Exclusive breastfeeding**		**Breastfeeding with occasional help**		**Breastfeeding mixed**		**Artificial breastfeeding**		**χ^2 (df)**	***p-Value***	***V***
	**Group**		**Group**		**Group**		**Group**				
	**IG *n* (%)**	**CG *n* (%)**	**IG *n* (%)**	**CG *n* (%)**	**IG *n* (%)**	**CG *n* (%)**	**IG *n* (%)**	**CG *n* (%)**			
Exclusive breastfeeding (n=298)	113 (78.5)	50(57.5)	16 (11.1)	10 (11.5)	8 (5.6)	14(16.1)	7 (4.9)	13(14.9)	16.08 (3)	<0.01	0.26
z-value	3.4	-3.4	-0.1	0.1	-2.6	2.6	-2.6	2.6			
Breastfeeding with occasional help(n=51)	9 (39.1)	4 (26.7)	2 (8.7)	1(6.7)	4 (17.4)	6 (40)	8 (34.8)	4(26.7)	2.41 (3)	0.5	0.25
z-value	0.8	-0.8	0.2	-0.2	-1.5	1.5	0.5	-0.5			
Breastfeeding mixed (n=33)	3 (23.1)	2 (28.6)	3 (23.1)	1 (114.3)	2 (15.4)	0 (0)	5 (38.5)	4 (57.1)	1.66 (3)	0.64	0.28
z-value	-0.3	0.3	0.5	-0.5	1.1	-1.1	-0.8	0.8			

Total type of breast-feeding postpartum	Type of breastfeeding at 6 months postpartum										
	**Exclusive breastfeeding**		**Breastfeeding with occasional help**		**Breastfeeding mixed**		**Artificial breastfeeding**		**χ^2 (df)**	***p-Value***	***V***
	**Group**		**Group**		**Group**		**Group**				
	**IG *n* (%)**	**CG *n* (%)**	**IG *n* (%)**	**CG *n* (%)**	**IG *n* (%)**	**CG *n* (%)**	**IG *n* (%)**	**CG *n* (%)**			
Exclusive breastfeeding (n=298)	87(65.9)	43(53.1)	14(10.6)	8(9.9)	26(19.7)	22(27.2)	5(3)	8(9.)	5.68(3)	0.12	0.12
z-value	1.9	-1.9	0.2	-0.2	-1.3	1.3	-1.8	1.8			
Breastfeeding with occasional help(n=51)	8(57.1)	1(8.3)	2(14.3)	3(25)	3(21.4)	8(66.7)	1(7)	--	8.81(3)	0.03	0.58
z-value	2.6	-2.6	-0.7	0.7	-2.3	2.3	0.9	-0.9			
Breastfeeding mixed (n=33)	3(37.5)	2(66.7)	--	--	4(50)	--	1(12.5)	1(33.3)	2.42(2)	0.30	0.
z-value	-0.9	0.9	--	--	1.5	-1.5	-0.8	0.8			

Note: χ^2, chi-square test; df, degrees of freedom; *V*, Cramer's *V*; significant p-Values < 0.05; IG, intervention group; CG, control group.
